# Examining Sensory Systems That Contribute to Falls in Parkinson Disease Using Computerized Dynamic Posturography: Secondary Analysis

**DOI:** 10.2196/84659

**Published:** 2026-05-07

**Authors:** Monica Rivera

**Affiliations:** 1 Department of Physical Therapy, California State University, Fresno, 5315 N Campus Drive MS/PT29, Fresno, CA, 93740, United States, 1 559-298-2052, 1 559-298-2625

**Keywords:** falls, sensory organization test, Parkinson disease, computerized dynamic posturography, sensory reweighting

## Abstract

In a retrospective study of 40 individuals with Parkinson disease, lower visual equilibrium scores (as measured by the sensory organization test, condition 2) were significantly associated with a higher frequency of falls.

## Introduction

Parkinson disease is a progressive neurological disorder characterized by a complex interaction of motor and nonmotor deficits. While it is established that motor impairments contribute to postural instability, a significant gap remains in understanding the role of sensory systems and their correlation to falls [1].

A core component of postural control is multisensory integration: the nervous system’s process of combining and reweighting sensory inputs. This mechanism prioritizes the most reliable sensory system to produce an accurate postural response [2,3]. Although the precise neuronal network is not fully understood, its purpose is to reduce reliance on inaccurate or less essential sensory inputs and increase weight to other systems to maintain stability [4,5]. This phenomenon is relevant in individuals with Parkinson disease, as they exhibit an increased reliance in the visual system to aid in everyday activities [6].

A component of computerized dynamic posturography—the sensory organization test (SOT) assesses how the visual, vestibular, and somatosensory systems contribute to postural control. This device examines postural sway across six diverse sensory conditions. Each condition isolates a specific sensory system and uses force plates to measure center of pressure displacements. These measures are then calculated into equilibrium scores. SOT conditions 1‐3 are static in nature: condition 1 measures quiet stance; condition 2 assesses postural control with eyes closed; and condition 3 distorts vision using optic flow. In contrast, condition 4 introduces a sway reference platform (ie, platform adjusts with center of pressure displacement) with eyes open; condition 5 consists of eyes closed and the sway reference platform (ie, vestibular system); and condition 6 distorts vision and implements the sway reference platform. Additional SOT variables include composite score, as well as visual, vestibular, and somatosensory ratios.

Although there is an extensive body of literature on falls in people with Parkinson disease, few studies have used the SOT to pinpoint specific sensory deficits. An early investigation by Bohnen identified the vestibular ratio score as an independent predictor of decreased postural control [7]. Subsequent studies have supported this finding, frequently citing SOT condition 5 as a primary factor in falls [8,9].

While investigations have identified the vestibular system as a key finding, subsequent research reveals similar findings in SOT conditions 1‐3. This finding links fall risk in community-dwelling adults to deficits in the visual system [10]. Given the uncertainty surrounding sensory system contributions, this study hypothesizes that specific sensory variables as measured by the SOT, are significantly correlated with and predictive of falls in individuals with Parkinson disease.

## Methods

### Study Design

This retrospective study compiled demographics, SOT condition scores, sensory ratios, and composite scores from three studies in individuals with Parkinson disease. In all three studies, recruitment and data collection protocols were consistent: SOT data were collected during the initial visit, and participants reported the number of falls in the preceding two-month period. Spearman rank correlation coefficients were calculated to determine the associations between SOT variables and reported falls. The largest correlation variables were entered into a multiple linear regression model to identify the most significant predictors of falls.

### Ethical Considerations

The studies comprising this study were approved by the Department of Physical Therapy and the University Human Subjects Review Board (Protocol Nos. 866, amended 866, and 1236). Each participant reviewed and signed an informed consent form prior to participation. This research did not receive external sponsorship or funding.

## Results

Forty participants (25 men and 15 women) were included in the analysis, with a mean age of 68.9 (SD 8.1) years. The reported fall data showed that 18 participants had experienced no falls, 12 participants had a history of 1 to 3 falls, and 10 participants had 4 to 6 falls. An a priori power analysis with a calculated moderate effect size (Cohen *d*=0.5), a desired power of 80%, and an α level of .05 determined a sample size of 42 participants.

Spearman rank correlation analysis showed a significant negative correlation for condition 1 (*r_s_*=−0.331; *P*=.04; ρ=.33, 95% CI −0.6 to 0), condition 2 *(r_s_*=−0.512; *P*<.001; ρ=−.51, 95% CI −0.73 to .021), and condition 3 *(r_s_*=−0.462; *P*=.003; ρ=−.46, 95% CI −0.39 to 0.15), indicating that lower scores in these conditions are associated with a higher frequency of reported falls.

Based upon the correlation results, SOT conditions 2 and 3 were selected as the predictors for the multiple regression model. The overall model was statistically significant, *F*_2,37_=7.05, *P*=.003, *R*^2^=.27, representing a large global effect size ƒ^2^=0.37 according to Cohen guidelines for regression models. Condition 2 was identified as the significant independent predictor of reported falls (*β*=−.23*, P*=.03), yielding a local effect size of *f ^2^*=0.14 indicative of a small to medium effect (Table 1).

**Figure 1.**
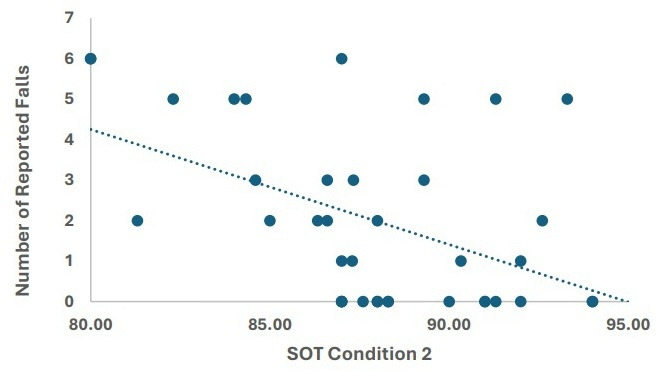
Sensory organization test (SOT) – condition 2 performance correlates with increased frequency of predicted falls in individuals with Parkinson disease (N=40).

The linear model for SOT condition 2 (predicted falls = 25.62 − (0.23 × SOT 2)) indicates that for every one-unit increase in the SOT 2 score, the predicted number of falls decreased by 0.23 (Figure 1).

**Table 1.** Predictors of fall among individuals with Parkinson disease: sensory organization test (SOT) regression (N=40).

Predictor	Unstandardized *β*	*P* value	95% CI
(Constant)	25.62	<.001	11.33, 39.92
SOT 2	−.23	.03	–0.44, –0.02
SOT 3	−.04	.42	–0.14, 0.06

## Discussion

This study highlights the complexity of sensory deficits in Parkinson disease by identifying reduced equilibrium scores in SOT condition 2 as a significant predictor of real-world falls. Although SOT condition 5 presents a greater postural challenge by distorting both visual and somatosensory inputs, falls in this condition occur within a simulated environment. Our findings suggest that condition 2, which tests balance during visual deprivation, is a more robust predictor of actual falls. Thus, as the disease progresses, individuals are likely to develop a visual dependency to compensate for sensory deficits. When visual input is compromised, the vestibular and somatosensory systems fail to effectively upweight their responses, thereby increasing fall risk.

Future research should employ a longitudinal, prospective design to track these variables over time, which would enable a more robust analysis of causality and reduce the risk of recall bias.

## Abbreviations

PD: Parkinson disease
SOT: sensory organization test
